# Stress-responsive gene regulation conferring salinity tolerance in wheat inoculated with ACC deaminase producing facultative methylotrophic actinobacterium

**DOI:** 10.3389/fpls.2023.1249600

**Published:** 2023-09-13

**Authors:** Kamlesh K. Meena, Ajay M. Sorty, Utkarsh Bitla, Akash L. Shinde, Satish Kumar, Goraksha C. Wakchaure, Shrvan Kumar, Manish Kanwat, Dhananjaya P. Singh

**Affiliations:** ^1^ Division of Integrated Farming System, Indian Council of Agricultural Research (ICAR)-Central Arid Zone Research Institute, Jodhpur, India; ^2^ School of Soil Stress Management, Indian Council of Agricultural Research (ICAR)-National Institute of Abiotic Stress Management, Baramati, India; ^3^ Department of Environmental Science–Environmental Microbiology, Aarhus University, Roskilde, Denmark; ^4^ Department of Biochemistry, Indian Council of Agricultural Research (ICAR)-Directorate of Onion and Garlic Research, Pune, India; ^5^ Indian Council of Agricultural Research (ICAR)-Crop Improvement Division, Indian Institute of Vegetable Research, Varanasi, India

**Keywords:** salinity stress, ACC deaminase, *Nocardioides*, methylotrophic bacteria, mitigation, wheat, ABA, Mitigation

## Abstract

Microbes enhance crop resilience to abiotic stresses, aiding agricultural sustainability amid rising global land salinity. While microbes have proven effective via seed priming, soil amendments, and foliar sprays in diverse crops, their mechanisms remain less explored. This study explores the utilization of ACC deaminase-producing *Nocardioides* sp. to enhance wheat growth in saline environments and the molecular mechanisms underlying *Nocardioides* sp.-mediated salinity tolerance in wheat. The *Nocardioides* sp. inoculated seeds were grown under four salinity regimes *viz.*, 0 dS m^−1^, 5 dS m^−1^, 10 dS m^−1^, and 15 dS m^−1^, and vegetative growth parameters including shoot-root length, germination percentage, seedling vigor index, total biomass, and shoot-root ratio were recorded. The *Nocardioides* inoculated wheat plants performed well under saline conditions compared to uninoculated plants and exhibited lower shoot:root (S:R) ratio (1.52 ± 0.14 for treated plants against 1.84 ± 0.08 for untreated plants) at salinity level of 15 dS m^−1^ and also showed improved biomass at 5 dS m^−1^ and 10 dS m^−1^. Furthermore, the inoculated plants also exhibited higher protein content *viz.*, 22.13 mg g^−1^, 22.10 mg g^−1^, 22.63 mg g^−1^, and 23.62 mg g^−1^ fresh weight, respectively, at 0 dS m^−1^, 5 dS m^−1^, 10 dS m^−1^, and 15 dS m^−1^. The mechanisms were studied in terms of catalase, peroxidase, superoxide dismutase, and ascorbate peroxidase activity, free radical scavenging potential, *in-situ* localization of H_2_O_2_ and superoxide ions, and DNA damage. The inoculated seedlings maintained higher enzymatic and non-enzymatic antioxidant potential, which corroborated with reduced H_2_O_2_ and superoxide localization within the tissue. The gene expression profiles of 18 stress-related genes involving abscisic acid signaling, salt overly sensitive (SOS response), ion transporters, stress-related transcription factors, and antioxidant enzymes were also analyzed. Higher levels of stress-responsive gene transcripts, for instance, *TaABARE* (~+7- and +10-fold at 10 dS m^−1^ and 15 dS m^−1^); TaHAk1 and hkt1 (~+4- and +8-fold at 15 dS m^−1^); antioxidant enzymes *CAT*, *MnSOD*, *POD*, *APX*, *GPX*, and *GR* (~+4, +3, +5, +4, +9, and +8 folds and), indicated actively elevated combat mechanisms in inoculated seedlings. Our findings emphasize *Nocardioides* sp.–mediated wheat salinity tolerance via ABA-dependent cascade and salt-responsive ion transport system. This urges additional study of methylotrophic microbes to enhance crop abiotic stress resilience.

## 1 Introduction

Agricultural productivity is vulnerable to ever-intensifying abiotic stresses under the current scenario of climate change. Salinization of agricultural lands is becoming one of the greatest threats to global crop yields. Attempts made to improve the agricultural losses due to salinity stress through breeding and genetic improvement programs have met limited success and are significantly time, cost, and labor intensive. Hence, a need to develop new strategies based on utilizing microbial capacities to strengthen biosaline agriculture has been proposed. Despite the significant progress already made in the area of microbial management of salinity stresses in crop plants (Kumar and Verma, 2018), there exists a bottleneck on wider applicability of plant biologicals under saline conditions typically due to underlying mechanisms of microbe-mediated salinity tolerance in plants. Majority of the currently available microbial inputs originate from different agricultural ecosystems, wild, stress-prone habitats, or from the plant’s phyllosphere region where the microbes form native communities (Dong et al., 2019; Xu et al., 2022) and are involved in beneficial plant interactions leading to stress tolerance (Inbaraj, 2021); however, their efficiency significantly differs due to differing mechanisms of action. For instance, the facultative methylotrophic bacteria from epiphytic habitat utilize single carbon (C1) substrates and produce a range of plant beneficial biomolecules including growth hormones (Meena et al., 2012). Inherent resilience of the methylotrophic bacteria merit them to thrive in constantly changing stressful microhabitat on the plant surface, particularly on the leaves, where methanol is abundant (Srivastava et al., 2022). Therefore, due to the characteristic resilience and beneficial bio-molecules production, methylotrophs make an important tool to maintain plant fitness under stressful conditions. Furthermore, inoculation of resilient microbes in agro-ecosystem is advantageous owing to their higher probabilities of survival and establishment, which reduces the chances of inoculum failure. Therefore, methylotrophic bacteria are of major interest in biosaline agriculture (Meena et al., 2020). Additionally, physiology of methylotrophic bacteria aligns with the criteria for proposed microbe-based stress management in plants (Meena et al., 2017; Munir et al., 2022). For instance, supra-optimal levels of ethylene under stress conditions could be efficiently regulated through 1-aminocyclopropane-1-carboxylic acid (ACC) deaminase, (Gupta and Pandey, 2019; Riyazuddin et al., 2020) while simultaneously produced growth hormones could sustain the plant growth and development.

Synthesis of ACC deaminase enzyme seems to be a rare characteristic among the known methylotrophic bacteria, with a few examples available such as *Methylobacterium fujisawaense* and *M. oryzae* (Madhaiyan et al., 2004). A positive influence of phytohormones producing and nitrogen fixing methylotrophs on plant growth and development is also documented (Fedorov et al., 2011; Meena et al., 2012). Consequently, such strains can have a high applicability as plant biologicals under stress conditions. However, despite the available knowledge on plant beneficial capabilities of methylotrophic bacteria (Ivanova et al., 2001; Subhaswaraj et al., 2017), their applicability as plant biologicals is limited due to the lack of knowledge on their specific plant interactions under stress conditions. Therefore, this study was designed to unfold the mechanistic interactions of wheat and an ACC deaminase producing methylotrophic bacterium under saline conditions. The major emphasis was to investigate post-methylotrophs inoculation changes at physicochemical and molecular level involving expression profile of stress-responsive enzymes and genes conferring salinity tolerance in wheat.

## 2 Materials and methods

### 2.1 Description of bacterial strain

The bacterial strain used in the study was originally isolated from soybean leaf and was identified as *Nocardioides* sp. by 16S rRNA gene sequencing (LC140963). The details of leaf-epiphytic methylotrophic bacterium *Nocardioides* sp. and its functional characteristics have been described earlier (Meena et al., 2020).

### 2.2 ACC deaminase activity

ACC deaminase activity was measured by the method described by Honma and Shimomura (1978), where the quantitation was achieved using a standard curve of the deamination product α-ketobutyrate in the range 0.1 µM to 1.0 µM. Briefly, the *Nocardioides* strain was initially grown in Luria Broth medium. The overnight grown cells were harvested, washed with sterile phosphate buffer saline (pH 7.0), and inoculated into the DF (Dworkin and Foster’s) medium (Dworkin and Foster, 1958) having ACC as sole nitrogen source and allowed to grow until late log phase was achieved. The cells were harvested, washed with tris-HCl (100 mM; pH 7.6), added with 5% toluene (v/v), and finally vortexed for 30 s to labilize the cells (Senthilkumar et al., 2021). The 50 μL of labilized cell suspension was then incubated with 5 μL of 0.3 M ACC for 30 min. The labilized cell suspension without ACC served as a negative control, while 0.1 M tris-HCl (pH 8.5) with 5 μL of 0.3M ACC served as a blank. The cell suspension was mixed thoroughly with 500 μL of 0.56N HCl by vortexing and cell debris were removed by centrifugation at 12,000 rpm for 5 min, a 500 μL of which was then mixed with 400 μL of 0.56 N HCl and 150 μL of DNF (2,4-dinitrophenylhydrazine in 100 ml of 2 N HCl) solution and incubated at 28°C for 30 min. One milliliter of 2N NaOH was added to the sample and the absorbance was recorded at 540 nm using a double beam spectrophotometer. The lysed cell suspension was used for estimation of protein content using Bradford assay (Bradford, 1976). Finally, the activity was calculated in terms of the nanomoles of α-ketobutyrate formed per milligram of protein in 1 hr.

### 2.3 Preparation of inoculum and impact assessment on wheat

The strain was cultivated in DF medium with ACC as nitrogen source at 30°C ± 2°C at 150 rpm for 72h. The cells were pelleted at 7830 rpm using centrifugation (Eppendorf *5430R*) and were washed with sterile phosphate-buffered saline (PBS), re-suspended in sterile PBS to obtain a CFU equivalent of 10^9^ ml^−1^. The inoculum was then used for priming wheat seeds.

Efficiency of the strain to mitigate salinity stress in wheat was evaluated in a gnotobiotic pot-experiment. Wheat seeds (*var. Lok*I) were treated with 5% sodium hypochlorite solution (Sauer et al., 1986) followed by several times washing with sterile milli-Q water to remove residual hypochlorite. The seeds were then treated with the inoculum (~10^9^cfu ml^−1^) in 0.1% carboxymethyl cellulose (CMC), dried aseptically in a laminar hood and finally sown into the pots filled with autoclave-sterilized garden-soil pre-adjusted to salinity levels of 0 dS m^−1^, 5 dS m^−1^, 10 dS m^−1^, and 15 dS m^−1^, respectively using crude salt (NaCl) solution. The pots were kept in dark for 72h at 20°C followed by 12h dark and light cycle till 28 days. Uninoculated seeds served as control.

### 2.4 Phenotypic characteristics of the wheat seedlings

The germination parameters *viz*. shoot-root length, germination percentage, seedling vigor index, total biomass, and shoot-root ratio were obtained from the freshly harvested 28 days old seedlings.

### 2.5 Biochemical analysis of wheat seedlings

#### 2.5.1 Preparation of enzyme extract

Enzyme extract was prepared from freshly harvested tissues by crushing in ice-cold phosphate buffer (100 mM; 0.5 mM ethylene diamine tetraacetic acid (EDTA); pH 7.5). Tissue debris was removed by centrifugation at 14000 rpm/10 min. The supernatant so obtained was stored at −20°C for further use as crude enzyme, and an aliquot of the extract was also used to determine the protein content. Ascorbic acid (1.0 mM) was added to the buffer while preparing the enzyme extract for determination of ascorbate peroxidase (Nakano and Asada, 1981).

#### 2.5.2 Antioxidant enzymes activity

Superoxide dismutase activity in the enzyme extract was assayed by measuring its ability to inhibit the photochemical reduction of Nitroblue tetrazoliumn by the method of Dhindsa et al. (1981). Briefly, 3 mL of reaction cocktail contained methionine (13.33 mM), EDTA (0.1 mM), phosphate buffer (50 mM, pH 7–8), sodium carbonate (50 mM), nitro blue tetrazolium chloride (NBT) (75 µM) and crude enzyme extract (10.0 µL) was added with riboflavin (2.0 µM). The reaction was started by illuminating the reaction mixture for 10 min under two white fluorescent lamps (15 W each). The reaction was stopped by shifting the reaction mixture in dark for 15 min. The absorbance was measured at 560 nm. A non-illuminated reaction mixture with no color served as control. The reaction mixture with no enzyme extract developed the maximum color, which decreased with the increase in the amount of added enzyme extract. Volume of enzyme extract corresponding to 50% inhibition reaction was considered as one enzyme unit.

Catalase activity was determined by monitoring the decrease in A_240_ resulting from the elimination of H_2_O_2_ as per the method described by Luck (1971). The standard reaction mixture contained (3.0 mL) potassium phosphate buffer (50 mM, pH 7.0), H_2_O_2_ (12.5 mM) and crude enzyme extract (50 µL). The decrease in absorbance was noted every 30s up to 3 min. The extinction coefficient of H_2_O_2_ at 240 nm was assumed to be 0.036 µm/ml. One unit (U) of the catalase activity was defined as the amount of enzyme required to degrade 1 µM mL^−1^ of H_2_O_2_.

Peroxidase activity was calculated according to Putter (Putter, 1974). Briefly, 3.0 mL of reaction mixture contained potassium phosphate buffer (50 mM; pH 6.1), guaiacol (16 mM), H_2_O_2_ (2.0 mM), and the crude enzyme extract (100 µL). The rate of formation of oxidized guaiacol in the guaiacol assay was measured at 436 nm.

Ascorbate peroxidase activity was estimated as described by Nakano and Asada (1981). Three milliliter of reaction mixture containing potassium phosphate buffer (50 mM; pH 7.0), ascorbic acid (0.5 mM), EDTA (0.1 mM), H_2_O_2_ (0.1 mM), and the enzyme extract (100 µL) was prepared. The reaction kinetics was monitored in terms of decreasing absorbance at 290 nm for 30 s. The oxidation of ascorbate was followed by the decrease in absorbance at 290 nm. The enzyme activity was calculated as μmol of ascorbic acid decomposed per minute with the absorbance coefficient 2.8 mM^−1^ cm^−1^ at 290 nm.

#### 2.5.3 Total sugars

The total sugar content from shoot and roots of the seedlings was estimated using anthrone reagent (Yemm, 1954). Briefly, 50 mg of sample was treated with 2.5M HCl in boiling water bath for 3h. The acid was neutralized with excess sodium carbonate and diluted to 50 mL. The debris was removed by centrifugation at 7830 rpm and 1.0 mL of supernatant was mixed with 4.0 mL of freshly prepared, ice-cold anthrone reagent (0.2% anthrone in 95% H_2_SO_4_). The contents were mixed thoroughly and kept in boiling water bath for 10 min, cooled under ambient condition, and read at 630 nm in terms of glucose equivalents.

#### 2.5.4 Phenolic compounds

The content of phenolic compounds was determined according to Singleton and Rossi (1965). For this, 100 mg of tissue samples were ground in 2.0 mL of chilled, 80% methanol. Debris was removed by centrifugation at 14000 rpm for 15 min, and 100 µL from the saved supernatant was mixed with Folin Ciocalteau reagent (1.0 N) under alkaline conditions. The reaction mixture was kept in boiling water bath for 1.0 min, cooled, and read at 650 nm. The Catechol was used as standard, and results were expressed in terms of mg CE g−^1^ fresh weight.

#### 2.5.5 Free radicle scavenging activity

Free radicle scavenging activity (FRSA) of the extract was estimated using 2,2-diphenyl-1-picrylhydrazyl (DPPH) radicle assay. Briefly, 1.0 ml aliquot of methanolic extract was mixed with 4.0 ml of DPPH reagent (40 µg ml^−1^ in methanol), and the initial absorbance was recorded at 517 nm. The tubes were then kept in dark under ambient conditions for 30 min followed by recording the post-reaction absorbance. The DPPH dissolved in methanol with no added plant extract served as control. Radical scavenging activity was expressed in terms of % antioxidant activity based on the decrease in absorbance at 517 nm using the formula-


$$\%\;antioxidant\;activity=\;\frac{Abs\left(control\right)-Abs\;\left(sample\right)}{Abs\left(Control\right)}\times \;100$$

#### 2.5.6 Total proteins

Protein content from the tissue was measured according to Bradford (1976). The tissue extracts obtained for enzyme estimation were mixed with Bradford reagent, allowed to react in dark for 15 min, and the absorbance was recorded at 595 nm. Proteins were quantified using the standard curve of bovine serum albumin.

#### 2.5.7 2-Deoxyribose degradation assay

The hydroxyl (OH) radical-scavenging activity plant extract was measured using deoxyribose degradation assay modified by Li et al, (2013) to assess the extent of DNA damage. Briefly, the shoot samples were washed with distilled water and shade dried at 40°C. The dried samples were extracted with 100% ethanol, filtered and vacuum dried. The extracts were solubilized in dimethyl sulfoxide and diluted with sterile water to obtain varying concentrations. Hydroxyl radical were generated by adding H_2_O_2_ (1 mM) to the 1 ml of final reaction mix containing phosphate buffer (pH 7.2) (25 mM), EDTA (100 μM), FeCl_3_ (100 μM), 100 µl DNA (3 mM), and varying concentration of plant extract. Reaction mix was incubated at 37°C for 1h, followed by mixing with 100 μl of 2% trichloroacetic acid (TCA) and 100 μl of 1% thiobarbituric acid (TBA) and heating for 15 min at 100°C. The absorbance was read at 532 nm. Hydroxyl radical inhibition potential was expressed in terms of “%inhibition” (Li, 2013) using the formula -


$$\%\;inhibition=\;\frac{Ao-A}{Ao}x\;100$$

Here, Ao is the absorbance of reaction mixture without plant extract and A is the absorbance of reaction mixture added with plant extract.

#### 2.5.8 Localization of oxidative radicles in wheat leaves

Localization of H_2_O_2_ and superoxide radicles in the leaves was analyzed using specific high-affinity dye. H_2_O_2_ localization was determined according to the method of Thordal-Christensen et al. (1997) with few modifications. Briefly, fresh leaves were immersed in freshly prepared 3,3′-diaminobenzidine (DAB) reagent (1.0 mg mL^-1^ DAB; 0.05% v/v Tween 20 and 10 mM Na_2_HPO_4_) and were subjected to vacuum infiltration for 15 min followed by leaving them for overnight for adequate staining. After decolorizing the leaves multiple times in ethanol:aceticacid:glycerol (3:1:1; v/v) in water bath for 10 min to remove chlorophyll, the decolorized leaves were kept on 60% glycerol-bed. Development of dark brown stain indicated the localization and distribution of reactive oxygen species (ROS) in the leaf tissues.

Similarly, localization of superoxide radicle was studied using NBT reagent (Jabs et al., 1996). Freshly harvested leaves were immersed in NBT reagent (0.2% NBT in 50 mM phosphate buffer; pH 7.5). Vacuum infiltration, decolorization, and the process of observation were identical to that of DAB staining.

### 2.6 Gene-expression profiling of the wheat seedlings

Freshly harvested tissues were immediately frozen in liquid nitrogen and subjected to RNA extraction using RNeasy plant mini kit (Qiagen, the Netherlands) following the manufacturer’s instructions. Immediately, synthesis of cDNA was done using Verso cDNA synthesis kit (Thermo Fisher Scientific, USA) as per manufacturer’s instructions.

Reference and target genes were quantitatively amplified in a 96-well cfx96 real-time PCR system (BioRad, USA), using DyNAmo Color Flash SYBR Green qPCR master mix (Thermo Fisher Scientific, USA). PCRs were carried out in a total volume of 20 µl containing 800 ng of cDNA, and 400 nM of each primer along with 1× of DyNAmo Colo rFlash SYBR Green qPCR master mix. Each reaction was carried out in three technical replicates along with a blank with sterile PCR-water serving as no template control (NTC). The reaction program in CFX manager (Version 3.1.1517.0823) consisted of 2 min at 50°C; 10 min at 95°C; 40 cycles of 30 s at 95°C and 1 min at annealing temperature of 60°C and 30 s at 72°C.

Melting curves were plotted through the range 65°C –95°C during which the fluorescence was recorded in steps of 0.5°C. The actin (*Act*) and beta-tubulin (β-tubulin) genes served as reference gene for normalization of the real-time qPCR data. The fold-change expression of transcript accumulation of target genes was quantified using the comparative 2^−ΔΔCt^ method. The primers used for quantification of 18 stress-responsive genes are depicted in the (**Supplementary Table S1**).

### 2.7 Statistical analyses

All the data were presented in mean values. All the numerical data were statistically analyzed using analysis of variance (ANOVA) with *post-hoc* Duncan’s multiple range test (DMRT), principal component, and clustering analysis was performed using SPSS 16.0 (Windows 8.0; SPSS Inc., www.spss.com/), Plotly (https://www.statskingdom.com/), and R 3.6.1 (Windows 10; www.cran.r-project.org/). The differences at the 95% confidence level were considered significant.

## 3 Results

The *Nocardioides* sp. (LC140963) exhibited positive reaction toward ACC deamination under *in vitro* conditions. The quantitative assay revealed the production of 1257 nM ± 72 nM of α-ketobutyrate mg^−1^ protein h^−1^. The scope of application of the actinobacterial strain was explored in this study through investigations on the mechanism of salt stress mitigation in wheat at phenotypic, biochemical, and molecular level. The results established a relationship among the physicochemical and molecular dynamics of wheat under saline conditions and mitigation induced by ACC deaminase producing bacterium.

### 3.1 Effect of bacterial application on physicochemical characteristics of wheat

**Figure 1:**
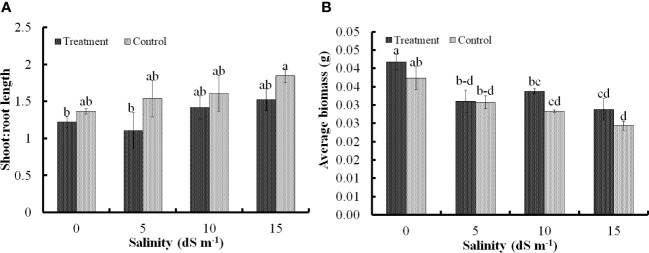
Impact of *Nocardioides* sp. on development of shoot and root of wheat seedlings in terms of shoot:root ratio **(A)**; and biomass accumulation **(B)** under saline conditions. Values in the same data series with different letters indicate significant difference (p less than or equal to 0.05) in the Duncan's multiple range test.

**Figure 2:**
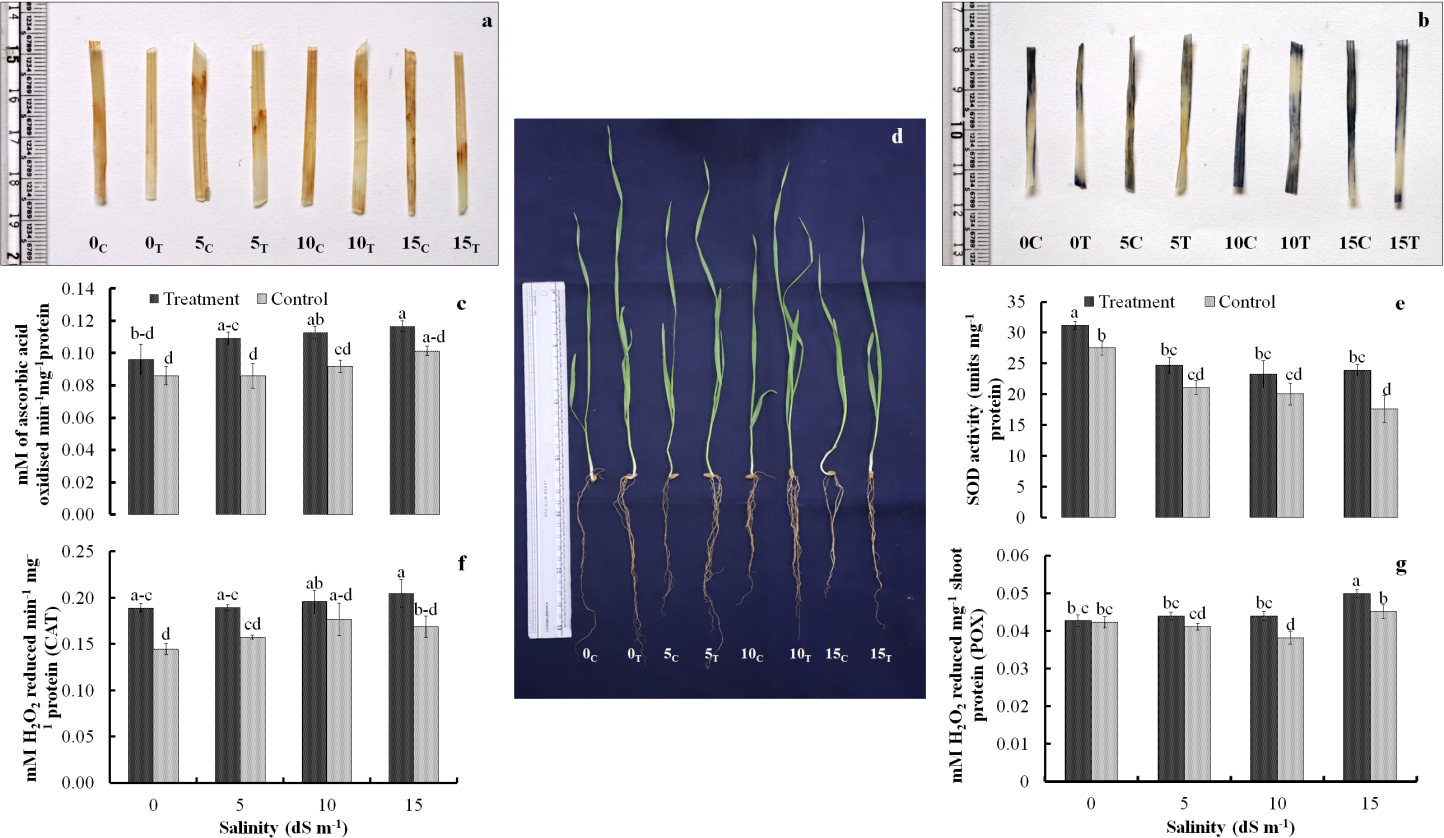
Localization of H_2_O_2_ radicals **(A)** and superoxide radicals **(B)** in treated and non-treated seedlings; trends of antioxidant enzymes ascorbate peroxidase **(C)**, superoxide dismutase **(E)**, catalase **(F)**, and peroxidase **(G)** activity as affected by salinity stress and *Nocardioides* sp. inoculation; and appearance of treated and untreated wheat seedlings under the influence of increasing salinity stress **(D)**. Values in the same data series with different letters indicate significant difference (p less than or equal to 0.05) in the Duncan's multiple range test.

Development of roots was influenced by the inoculation. Saline conditions indeed restricted root development, which was clearly evident from a higher S:R ratio; however, under the similar scenario, the inoculation of *Nocardioides* sp. significantly lowered the S:R ratio, thereby, confirming that the root development was successfully sustained from 5 dS m^−1^ to 15 dS m^−1^ (**Figures 1A**, **2D**). Lower S:R ratio of 1.52 ± 0.14 of the treated seedlings as against 1.84 ± 0.08 for the untreated ones at salinity level of 15 dS m^−1^. Root development was stimulated predominantly at low (5 dS m^−1^) and high (15 dS m^−1^) levels of salinity stress, although there was moderate improvement at 10 dS m^−1^ (**Figure 1A**). Similarly, the highest average biomass of 0.041 ± 0.03 g seedling^−1^ was recorded under non-saline conditions, but the bacterial application significantly improved the biomass at 5 dS m^−1^ and 10 dS m^−1^. However, the effect of bacterial application on biomass remained non-significant at 15dS m^−1^ (**Figure 1B**).

Biochemical parameters of wheat seedlings were also significantly influenced by the inoculation. Activity of oxidative stress management characteristics showed critical responsiveness toward the bacterial inoculation. Catalase, peroxidase, and ascorbate peroxidase activities revealed increasing trend with increasing salinity stress. Ascorbate peroxidase and catalase activity in inoculated seedlings increased mainly at higher salinity levels of 10 dS m^−1^ and 15 dS m^−1^ (**Figures 2C, F**). The peroxidase activity, however, remained almost steady up to 10 dS m^−1^ but eventually increased significantly at 15 dS m^−1^ (**Figure 2G**). The SOD activity declined markedly in control at 15 dS m^−1^ and remained stable at all the three stress levels with peak activity in seedlings with no stress (0 dS m^−1^) (**Figure 2E**). In treated groups also, the SOD activity remained almost stable at all the salinity stress levels. The *in-situ* localization of H_2_O_2_ and O^+^radicals was also likely to be linked to the activity of oxidative stress management machinery (**Figures 2A, B**). Elevated levels of oxidative radicals were noted in control compared to the treated seedlings especially making substantial difference at higher salinity stress levels of 10 dS m^−1^and 15 dS m^−1^.

Free radical scavenging activity of seedling extract, as evaluated by DPPH assay, showed effective neutralization of the free radicals by the extract. Among all the salinity stress levels (5 dS m^−1^ to 15 dS m^−1^), including no stress (0 dS m^−1^) the free radical scavenging activity of extract was higher in treated samples compared to the control, indicating stimulation of the antioxidant mechanism in the inoculated wheat seedlings (**Table 1**). The effect of inoculation on antioxidant activity was much higher in treated plants (26.68%) as compared to control (21.67%) at 5 dS m^−1^.

**Table 1:** Biochemical attributes of the wheat seedling tissues under the influence of *Nocardioides* sp.

Salinity (dS m^-1^)	Treatment	Protein^#^	Sugar^*^	Phenolics^£^	DPPH^†^	Deoxy ribose protection^‡^
0.0	Inoculated	**22.13 ± 1.87^ab§^**	24.97 ± 1.23^c^	2.99 ± 0.37^b^	**24.74 ± 1.12^ab^**	12.57 ± 2.44^d^
	Control	18.34 ± 2.11^c^	24.51 ± 0.73^c^	2.89 ± 0.19^b^	22.44 ± 2.02^bc^	13.26 ± 1.92^d^
5.0	Inoculated	**22.10 ± 1.87^ab^**	26.28 ± 1.19^c^	3.39 ± 0.66^ab^	**26.68 ± 2.02^bc^**	**21.08 ± 1.18^bc^**
	Control	20.53 ± 1.83^a-c^	24.67 ± 1.38^c^	3.15 ± 0.68^ab^	21.67 ± 1.50^cd^	18.47 ± 1.63^c^
10.0	Inoculated	**22.63 ± 1.96^ab^**	**30.33 ± 1.16^b^**	**3.83 ± 0.29^a^**	**24.63 ± 0.75^ab^**	**22.53 ± 1.76^ab^**
	Control	19.32 ± 1.85^bc^	25.75 ± 1.61^c^	3.19 ± 0.39^ab^	21.66 ± 1.48^cd^	17.92 ± 1.61^c^
15.0	Inoculated	**23.62 ± 1.83^a^**	**32.77 ± 1.34^a^**	**3.85 ± 0.47^a^**	**23.36 ± 1.60^bc^**	**25.31 ± 1.45^a^**
	Control	17.50 ± 1.79^c^	28.45 ± 1.06^b^	3.03 ± 0.44^ab^	19.22 ± 1.17^d^	20.43 ± 1.72^bc^

^#^Protein content.

^*^Total sugars.

^£^Total phenolic compounds expressed in mg g^−1^ fresh weight.

**^†^** Percent reduction of DPPH dye by the phenolic compounds from wheat seedlings.

^‡^Percent inhibition of 2-deoxy-D- ribose decomposition in presence of tissue extract of wheat seedlings.

^§^Values in the same data series superscripted by different letters indicate significant difference at p = 0.05 in Duncan’s multiple range test.

Significantly induced values are marked in bold.

Predominant biochemical traits comprising the contents of protein, sugars, and plant phenolics were assessed to generate an overview of cellular homeostasis of the inoculated seedlings. The protein content in inoculated seedlings remained significantly higher over the control with steady trend (22.13 mg g^−1^, 22.10 mg g^−1^, and 22.63 mg g^−1^ fresh weight, respectively) at 0 dS m^−1^, 5 dS m^−1^, and 10 dS m^−1^. However, a marked rise in protein content (23.62 mg g^−1^ fresh weight in inoculated plants compared to 17.50 mg g^−1^ fresh weight in control) at 15 dS m^−1^ was also observed (**Table 1**). The difference in sugar and plant phenolics content in treated and control remained statistically insignificant at 0 dS m^−1^ and 5 dS m^−1^, but sugar content was significantly increased at 10 dS m^−1^ and 15 dS m^−1^ (**Table 1**). The phenolic content in inoculated wheat seedlings was significantly higher under saline conditions (at 5 dS m^−1^, 10 dS m^−1^, 15 dS m^−1^ EC) compared to 0 dS m^−1^. The difference in plant phenolics content was insignificant for three salinity stress levels.

The ability of extracts to inhibit the degradation of 2-deoxy-D-ribose, a nucleotide sugar was also investigated as a protective indicator of the DNA damage. Except for the treatment at 0 dS m^−1^ the inoculated seedlings exhibited significantly higher ability to inhibit the hydroxyl radical-mediated degradation of 2-deoxy-D-ribose sugar in DNA, which was evident by higher % inhibition of 21.08%, 22.53%, and 25.31% recorded for inoculated seedling grown at 5 dS m^−1^, 10 dS m^−1^, and 15 dS m^−1^, respectively, as against the 18.47%, 17.92%, and 20.43% inhibition recorded for uninoculated control seedlings at similar salinity levels (**Table 1**). These results indicated the presence of more efficient mechanisms to protect salinity-induced DNA damage into the inoculated seedlings.

### 3.2 Localization of H_2_O_2_ and superoxide radicals

The DAB- and NBT-based histochemical staining assays were used for *in-situ* localization of H_2_O_2_ and superoxide radicals, respectively. The development of reddish and purple color patches indicated the presence of H_2_O_2_ and superoxide radicals in the leaf tissues. A greater number of radish color patches developed in the leaves of uninoculated control wheat seedlings at no salinity stress (0 dS m^−1^) and also at all salinity stress levels (5 dS m^−1^, 10 dS m^−1^, and15 dS m^−1^) (**Figure 2A**). Similarly, the localization of superoxide radicals was inferred by development of purple color patches in NBT staining. Higher abundance of purple color spots was seen in leaves of uninoculated control wheat plants at no salinity stress (0 dS m^−1^) and also at all salinity stress levels (5 dS m^−1^, 10 dS m^−1^, and 15 dS m^−1^) (**Figure 2B**). The sharp differences were observed in purple color development in inoculated and uninoculated plants at salinity level of 5 dS m^−1^ and 10 dS m^−1^. However, the results at higher salinity level of 15 dS m^−1^ were not so obvious.

### 3.3 qPCR-based quantification of wheat genes implicated under salinity stress

**Figure 3:**
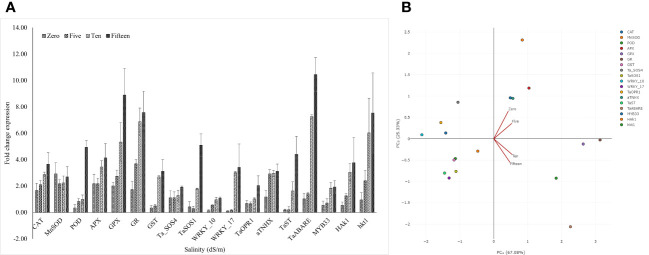
Status of different stress responsive gene transcripts at different salinity levels **(A)**. Principle component analysis of gene expression trends under saline conditions **(B)**. Note the relatively aligning trends of expression, respectively, under low intensity (0 dS m^−1^ to 5 dS m^−1^) and higher intensity (10 dS m^−1^ to 15 dS m^−1^) of salinity stress.

**Figure 4:**
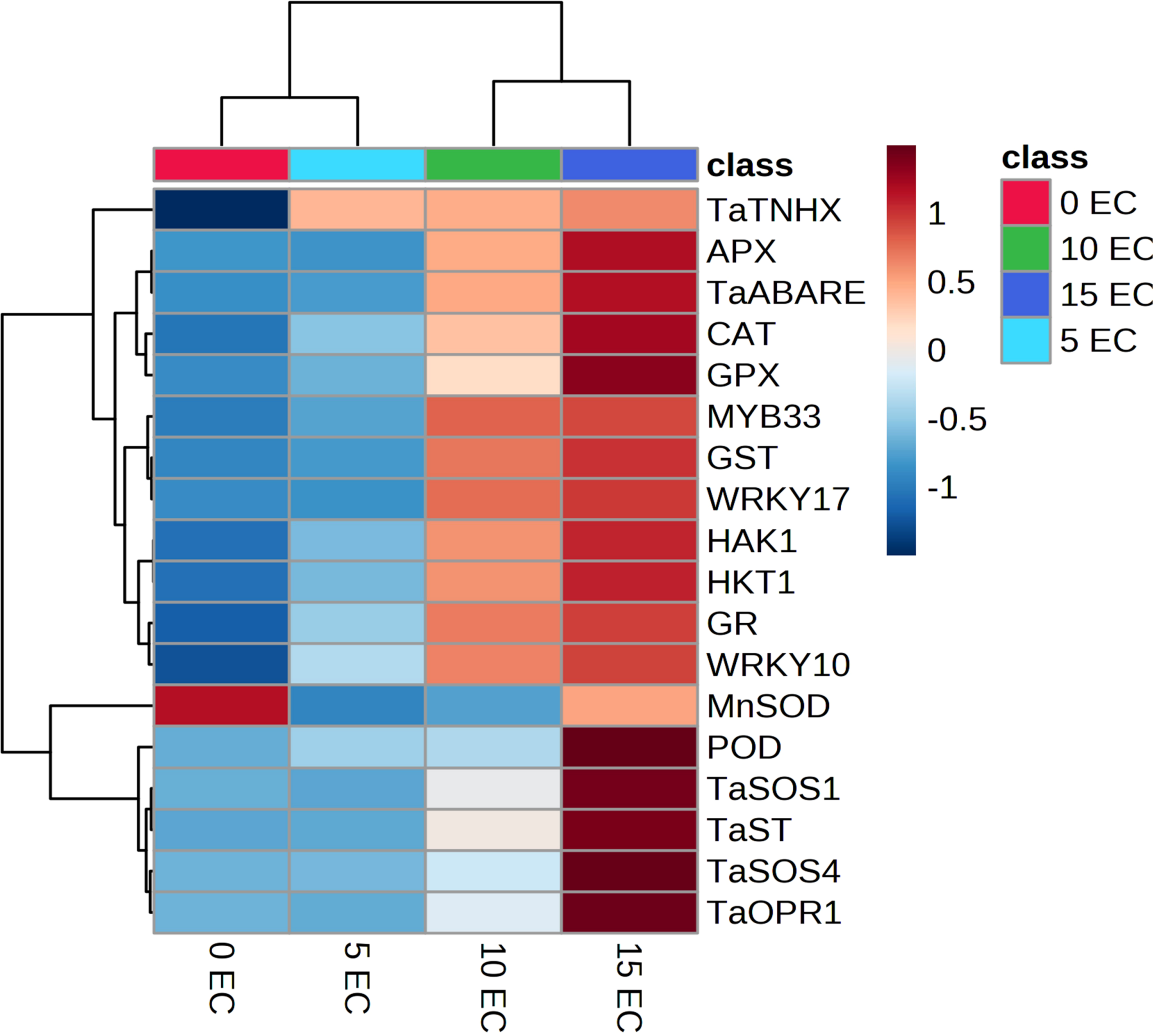
Clustered heat map showing specific expression trends of salt-responsive genes in wheat. Note the gradually increasing expression trends from no stress to highest stress regime.

We used qRT-PCR to study the expression of 18 genes known to confer plant stress-response against salinity conditions. These genes were related to abscisic acid signaling (*TaABARE* and *TaOPR1*), SOS response genes (*TaSOS1* and *TaSOS4*), and the genes for stress-related transcription factors (*TaWRKY10*, *TaWRKY17*, *TaMYB33*, and *TaST*), ion transporters (*TaHKT*, *TaNHX*, and *TaHAK1*), genes for antioxidant enzymes (*POD*, *CAT*, *MnSOD*, *APX*, *GR*, and *GPX*). **Figure 3A** details the trends of the studied gene transcripts at different salinity levels. The qPCR-based expression studies revealed around 7- and 10-fold higher expression of *TaABARE* gene in inoculated plants at higher salinity levels of 10 dS m^−1^ and 15 dS m^−1^, respectively compared to unstressed plants at 0 dS m^−1^. Minor differences were observed in *TaOPR1* transcripts of inoculated plants at all salinity levels. Similarly, the expression profiling of two genes (*TaSOS1* and *TaSOS4*) of salt overly sensitive (SOS) pathway showed around twofold and fivefold higher expression of *TaSOS1* at salinity stress of 10 dS m^−1^ and 15 dS m^−1^, respectively; however, change in gene expression of *TaSOS4* at all salinity stress levels was non-significant. Among the transcripts of the three-transcription factor-linked genes, namely, *TaWRKY10*, *TaWRKY17*, and *TaMYB33* showed a higher expression of *TaWRKY17* with the increasing salinity stress levels. The expression of *TaWRKY10* and *TaMYB33* also increased with increasing salinity stress, but the differences were non-significant for 10 dS m^−1^ and 15 dS m^−1^. The three ion transporter genes *TaHKT*, *TaNHX*, and *TaHAK1* also showed increased expression at higher salinity stress levels. The expression of *TaHAK1* and *HKT1* was fourfold and eightfold higher, respectively, in inoculated plants at 15 dS m^−1^. A higher expression of genes (*POD*, *CAT*, *MnSOD*, *APX*, *GR*, and *GPX*), coding antioxidant enzymes was observed in inoculated plants compared to uninoculated plants and the differences were more obvious at higher salinity (15 dS m^−1^), except for *MnSOD*, whose expression did not increase with salinity stress. The change in expression of GR and GPX was highest with eight and ninefold higher, respectively, in inoculated plants compared to their uninoculated counterparts at 15 dS m^−1^. Principal component analysis (PCA) vectors of the seedlings grown in 0 dS m^−1^ and 5 dS m^−1^ indicated relatedness in gene expression trends (**Figure 3B**). However, those of 10 dS m^−1^ and 15 dS m^-1^ remained significantly diverging in patterns. Thus, in general, a characteristic expression of salinity-induced genes appeared more prominent at the salinity levels above 5 dS m^−1^ (**Figure 3A**). The increasing expression trends typically above 5 dS m^−1^ indicated perception and mounting of indigenous responses by the plants. However, microbial amendment incorporated an additive effect to the indigenous plant responses, thereby improving the physiochemical performance of the seedlings over the control. The alignments of gene expression trends were further corroborated by specific clustering of 0–5; and 10–15 dS m^−1^ regimes (**Figure 4**).

## 4 Discussion

Many bacterial genera of phylum Actinobacteria, such as *Arthrobacter*, *Frankia*, *Streptomyces*, *Micromonospora*, *Micrococcus*, and *Kocuria*, are widely reported to exhibit plant growth promotion activities (Mitra et al., 2022). The application of BCFE (bacterial culture filtrate extract) derived from actinobacterial genus *Nocardioides* and its positive influence on wheat growth parameters under saline condition was reported in our earlier report (Meena et al., 2020). On similar lines, the *Nocardioides* sp. KNC-3 isolated from Moroccan sites showed the ability to grow on N-free medium indicative of its nitrogen fixing ability (Nafis et al., 2019). The antinobacterial methylotrophic strain *Nocardioides* sp. NIMMe6 used in this study was able to degrade ACC and thereby utilizing sole nitrogen source in the selective media (indicative of its ability to downregulate the ethylene production under stress conditions by sequestering and cleaving plant-produced ACC, which is the biochemical precursor for the ethylene biosynthesis, thereby sinking the excess ethylene to protect the plant from its inhibitory effect. Ethylene, being a gaseous hormone, is involved in plant response to diverse environmental stresses and its synthesis is reported to be induced under salinity stress conditions (Yang et al., 2009). Reduced ethylene levels lead to increased stress tolerance in plants to a wide range of environmental stresses (Glick, 2005). In our study, the inoculated seeds with *Nocardioides* sp. resulted in better root growth (lower S:R ratio) and a superior biomass at higher salinity levels compared to the uninoculated control, which indicated that the inoculated plants better tolerated the negative impact of salinity stress. The negative impact of higher ethylene level on root growth have also been reported in other crops like groundnut (Saravanakumar and Samiyappan, 2007) and *Pisum Sativum* (Barnawal et al., 2014, Gupta et al, 2021).

Exposure to salinity stress leads to increased ROS production in plants stimulating oxidative damage to cell membranes (Talaat, 2019). The ROS so produced are neutralized by non-enzymatic and enzymatic detoxification systems to protect the plants from oxidative damage (Zhang et al., 2022). In this study, the inoculated plants displayed a higher activity of antioxidant enzymes at all salinity levels compared to the uninoculated control plants indicating the positive influence of *Nocardioides* inoculation on enzymatic antioxidant system in wheat. The results of qPCR-based gene expression experiment also confirmed increased expression of CAT (encoding subunit of catalase), POD (gene encoding peroxidase), and APX (gene encoding for ascorbate peroxidase) genes under salinity stress conditions. The increase in gene expression levels CAT, POD, and APX suggest that the *Nocardioides* sp. inoculation resulted in effective scavenging of ROS species by increasing the *de novo* synthesis of antioxidant enzymes at transcription level. The increased activities of peroxidase (POD) and catalase (CAT) in plant growth promoting rhizobacteria (PGPR)-inoculated wheat plants are in agreement with previous report of Haroon and coworkers (Harron et al., 2021), where *B. tequilensis* inoculated plants showed significant increase in activity of POD and CAT at 100 mM salinity compared to uninoculated control. The *in-situ* localization of H_2_O_2_ and superoxide radicles was also confirmed by DAB reagent-based assay (Thordal-Christensen et al., 1997) and NBT-based assay (Jabs et al., 1996), respectively. Using both the free radicle localization assays, we showed the lower production of H_2_O_2_ and superoxide radicle in inoculated plants and, hence, supposedly leads to lesser damage to cellular membranes and, thus, leading to high salt stress tolerance in *Nocardioides* sp. inoculated plants. The higher expression of GPX gene (glutathione peroxidase gene) and GR (Glutathione reductase) was noted in inoculated plants at higher salinity level (10 dS m^−1^ to 15 dS m^−1^). Glutathione peroxidase catalyses the reduction of hydrogen peroxide (H_2_O_2_) to water (H_2_O), using glutathione (GSH) as a reducing agent. The GR gene encodes the glutathione reductase enzyme, which catalyzes the reduction of oxidized glutathione (GSSG) to reduced glutathione (GSH) using NADPH as a cofactor. The higher expression of GPX and GR genes at higher salinity stress (15 dS m^−1^) indicates the important role of ascorbate-glutathione antioxidant system in scavenging free radicles produced due to the salinity stress.

We also assessed the FRSA of extracted phenolic compounds using DPPH assay. Phenolic compounds are the most crucial non-enzymatic antioxidants for scavenging the excessive ROS that is generated during stress conditions (Kiani et al., 2021). Our results indicate higher phenolics content in inoculated wheat seedlings compared to the untreated control, and our results also corroborated well with higher DPPH scavenging activity observed in inoculated wheat seedlings. Among various types of ROS produced under stress, the hydroxyl radical (^•^OH) occupies an exceptional position because of its extreme reactivity and its ability to attack even the inert compounds like alkanes, which are otherwise considered to be stable under physiological conditions (Wilson et al., 2006). We used a modified deoxyribose degradation method to assess the DNA damage protection ability of wheat seedlings extract. The hydroxyl radicals were generated *in vitro* in the reaction mix by Fenton reaction and wheat seedling extracts were added to scavenge the hydroxyl radicals causing DNA damage. Our results indicated that the *Nocardioides* sp. inoculated plants showed a higher ability to scavenge hydroxyl radical (^•^OH), thereby minimizing the hydroxyl radical‐induced DNA damage under salinity stress conditions. The non-structural polyphenolic compounds and flavonoids are reported to be involved in scavenging the Hydroxyl radicals produced during stress conditions (Kaur et al., 2017). The hydroxyl radical scavenging activity in salt stressed wheat is reported to increase with addition of the exogenously supplied phenolic acids compared to the seedling grown without phenolics application. When plants are subjected to salt stress, one of the most common biological and physiological effects is an increase in the generation of ROS, which include hydroxyl radical (OH), superoxide radical (O_2_), and hydrogen peroxide (H_2_O_2_). Our observations pertaining to antioxidant enzymatic activity assay, DPPH assay and hydroxyl radical scavenging activity assay clearly show that the *Nocardioides* sp. inoculated plants effectively neutralized the free radicals produced and maintained a good redox homeostasis under saline conditions, thereby avoiding damage to cellular membranes and thus registered better growth parameters. These results also corroborated well with the results obtained for H_2_O_2_ and superoxide radical localization assays performed on intact wheat leaf tissue for inoculated and uninoculated control plants using DAB reagent and NBT reagent, respectively. The *in-situ* localization of free radicals clearly indicated that the accumulation of H_2_O_2_ and superoxide radical was much lower in inoculated plants even at higher salinity of 15 dS m^−1^ compared to the uninoculated control plants (**Figures 2A, B**). The DAB used for H_2_O_2_ localization polymerizes into a radish brown polymer immediately at sites of peroxidase activity as soon it comes into contact with H_2_O_2_ (Thordal-Christensen et al., 1997). The development of smaller and less prominent patches of radish color in treated inoculated plants at all salinity levels indicated that the production of H_2_O_2_was lesser in inoculated plants compared to uninoculated control. Similarly, the NBT staining was used to localize the superoxide radicals, whose presence was indicated by the development of purple precipitate in the target leaf tissue (**Figure 2B**). The lesser number of purple lesions developed in inoculated plants indicated lesser superoxide ions produced under salinity stress in *Nocardioides* sp. inoculated wheat plants. In addition to enhancing ROS scavenging abilities and antioxidant enzyme activities, the improved regulation of ion balance contributed to the enhanced salt tolerance observed in inoculated plants.

The increased expression of *TaSOS1* gene in inoculated plants at higher salinity implies better regulation of ion homeostasis through SOS pathway. The SOS pathway involves a series of signaling events that result in the activation of plasma membrane ion transporters, such as Na^+^/H^+^antiporter, which pumps excess Na^+^ ions out of the cell and helps to maintain a favorable cytosolic Na^+^/K^+^ ratio (Zhu, 2016). SOS1 is a plasma membrane Na^+^/H^+^ anti-porter, which plays a crucial role in regulating long-distance Na^+^ transport from root to shoot, contributing to salt tolerance. On the other hand, SOS4 encodes pyridoxal kinase, which is involved in the biosynthesis of pyridoxal-5-phosphate, an essential cofactor for numerous cellular enzymes (Nidhi et al., 2016). A higher expression of *TaTNHX*, HAK1, and *HKT1* genes which encodes for “vacuolar cation/proton exchanger,” “potassium transporter,” and “high-affinity sodium transporter,” respecti*v*ely was observed in inoculated plants compared to their uninoculated counterparts suggesting that the increased salt stress tolerance in inoculated plants was mediated through the increased activity of ion transporters. The over expression of *TaTNHX* has been shown to enhance salt tolerance in plants such as *Arabidopsis* by reducing the accumulation of toxic Na^+^ ions in the cytoplasm and thereby increasing the sequestration of Na^+^ ions into the vacuoles (Li et al., 2019). Similarly, the over expression of *HAK1* gene in *Arabidopsis thaliana* and bread wheat has been linked to improved salt tolerance in both plants (Li et al., 2021). The over expression of wheat HKT1 gene in transgenic alfalfa resulted in improved salt tolerance suggesting that the regulation of HKT1 expression and activity is a key factor in improving plant salt tolerance (Zhang et al., 2018). In this study, we also studied the expression profile of genes encoding for transcription factors such as WRKY10, WRKY17, and MYB33 in inoculated and uninoculated plants. Transcription factor genes are known to play a crucial role in the regulation of various stress-responsive genes that are involved in salt stress tolerance mechanisms in plants. We noted a higher expression of WRKY17 in inoculated plants at higher salinity suggesting its critical role in regulating salt stress-response in wheat seedlings. WRKY17 over expression was found to up regulate the expression of various stress-responsive genes, including those involved in ion transport and osmotic adjustment (Wang et al., 2019).

## 5 Conclusion

The results clearly underscore the applicability of methylotrophic bacteria for enhancing salinity stress tolerance in wheat. *Nocardioides* sp. used in this study implies major modes of action include ABA-mediated cascades, subsequent enhanced enzyme and non-enzyme-based oxidative stress management, cellular osmolytes, and important membrane proteins involved in ionic interactions to manage cellular Na^+^ levels. The transcript profiles indicate involvement of ABA as predominantly functioning pathway for *Nocardioides* sp.–mediated salt stress management in early wheat. With these newly available mechanisms, an investigation of the *Nocardioides* strain under naturally stressed conditions is required to validate its capabilities as a biological input to mitigate salinity (and potentially drought) stress in wheat.

## Data availability statement

The data presented in the study are deposited in the DDBJ-GenBank repository, and can be accessed at https://www.ncbi.nlm.nih.gov/nucleotide/ with the accession number LC140963.

## Author contributions

KM: conceptualization; KM and AMS: experimental design; AMS, UB, ALS, and SaK: experimental implementation; UB, AMS, GW, and SaK: results compilation and statistical analyses; AMS, KM, and UB: initial draft preparation; SaK, MK, ShK, and DS: copy editing and improvements. All authors contributed to the article and approved the submitted version.
