# Supplementary material for: Stress-responsive gene regulation conferring salinity tolerance in wheat inoculated with ACC deaminase producing facultative methylotrophic actinobacterium
Source: Front Plant Sci. 2023 Sep 13;14:1249600. doi: 10.3389/fpls.2023.1249600 (PMC10534068; doi:10.3389/fpls.2023.1249600)
Supplement: Supplementary file 1 [file Table_1.docx]

**Stress-responsive gene regulation conferring salinity tolerance in wheat inoculated with ACC deaminase producing facultative methylotrophic actinobacterium**

Kamlesh K. Meena*^1†^; Ajay M. Sorty^2,3†^; Utkarsh Bitla^2^; Akash L. Shinde^2^; Satish Kumar^4^; Goraksha C. Wakchaure^2^; Shrvan Kumar^1^, Manish Kanwat and Dhananjaya P. Singh^5^

**Supplementary Table S1 -** Details of primers used in study

| Gene | Primer | Sequence | Reference |
| --- | --- | --- | --- |
| ABARE(JK546530) | Forward | 5’-CGATGATGAACTCCACTCA-3’ | Garg et al. 2013 |
|  | Reverse | 5’-ATGATATAGTTCCAAAGG-3’ |  |
| TaWRKY17(JK546463) | Forward | 5`GACCAAGCGGCTCAACGAT-3' |  |
|  | Reverse | 5`GGTGCATATGCTAGCTTG-3' |  |
| POD(X56011) | Forward | 5'-CAGCGACCTGCCAGGCTTTA-3' | Jiang et al. 2012 |
|  | Reverse | 5'-GTTGGCCCGGAGAGATGTGG-3' |  |
| TaNHX | Forward | 5'-ATCTACYTSCTCCCKCCCAT-3' | Saqib et al. 2005 |
|  | Reverse | 5'-AGATAGCYCCAATTGCAAGA-3' |  |
| TaST (EF675609) | Forward | 5 ́-CGCAGGCCGTCGTCATG-3 | Huang et al. 2012 |
|  | Reverse | 5 ́-GACTGATCCTGCCAGCAAACAC-3' |  |
| TaMYB33(JN584645) | Forward | 5'-GTCGTCCGCCACAGATTACTC-3' | Qin et al. 2012 |
|  | Reverse | 5'-CGAACGATTATTGTTCCCTTCAC-3' |  |
| TaWRKY10(EF368361) | Forward | 5`AGCTCGTCTGTGCAGTGCACTTAT-3` | Li et al. 2013 |
|  | Reverse | 5`TCGTGTACATGCATCCGTGAGATT-3` |  |
| MnSOD(GI1622928) | Forward | 5'-CAGAGGGTGCTGCTTTACAA-3' | Baek and Skinner, 2003 |
|  | Reverse | 5'-GGTCACAAGAGGGTCCTGAT-3' |  |
| TaSOS1(AY326952) | Forward | 5'- GTTGTCGGTGAGGTCGGAGGG -3' | Ramezani et al. 2013 |
|  | Reverse | 5'- TCATCTTCTCCTACCGCCCTGC-3' |  |
| TaSOS4(AY337321) | Forward | 5'-ATCCAGTCCCACACCGTCCA -3' |  |
|  | Reverse | 5'- GCTGATTGCCATTGAGAACCTGTC-3' |  |
| TaOPR1(JQ409278) | Forward | 5'-ACTGCCACGACTCCGACCC-3' | Dong et al. 2013 |
|  | Reverse | 5'-CCGATGCGACCGCCTTG-3' |  |
| HAK1(JF495466) | Forward | 5’-ACGCTTACGGGATCTGTGTG-3’ | Bharti et al., 2016 |
|  | Reverse | 5’-GAGCCGAACACGACGTAGAA-3’ |  |
| hkt1(U16709) | Forward | 5’-ATGGGCCGGGTGAAAAGATT-3’ |  |
|  | Reverse | 5’-TCCAGAAGGGGTGAACATGC-3’ |  |
| CAT(GI5711144) | Forward | 5'-CCATGAGATCAAGGCCATCT-3' | Baek and Skinner 2003 |
|  | Reverse | 5'-ATCTTACATGCTCGGCTTGG-3' |  |
| GR(TC84151) | Forward | 5'-TGCGTCCCGAAGAAGATACT-3' |  |
|  | Reverse | 5'-GTTGATGTCCCCGTTGATCT-3' |  |
| APX(TC22268) | Forward | 5'-GCAGCTGCTGAAGGAGAAGT-3' |  |
|  | Reverse | 5'-CACTGGGGCCACTCACTAAT-3' |  |
| GPX(TC22467) | Forward | 5'-CCCCCTGTACAAGTTCCTGA-3' |  |
|  | Reverse | 5'-GTCAACAACGTGACCCTCCT-3' |  |
| Actin (AB181991) | Forward | 5'-CGAAACCTTCAGTTGCCCAGCAAT-3' | Dong et al. 2013 |
|  | Reverse | 5'-ACCATCACCAGAGTCGAGCACAAT-3' |  |
| β-Tub | Forward | 5'-CCGCGCTGTCTTTGTAGAT-3' |  |
|  | Reverse | 5'-CATCCTCCTTGCCACTGATAA-3' |  |

**References**

Baek, K. H., & Skinner, D. Z. (2003). Alteration of antioxidant enzyme gene expression during cold acclimation of near-isogenic wheat lines. Plant Science, 165(6), 1221-1227.

Bharti, N., Pandey, S. S., Barnawal, D., Patel, V. K., & Kalra, A. (2016). Plant growth promoting rhizobacteria *Dietzia natronolimnaea* modulates the expression of stress responsive genes providing protection of wheat from salinity stress. Scientific reports, 6(1), 34768.

Dong, W., Wang, M., Xu, F., Quan, T., Peng, K., Xiao, L., & Xia, G. (2013). Wheat oxophytodienoate reductase gene *TaOPR1* confers salinity tolerance via enhancement of abscisic acid signaling and reactive oxygen species scavenging. Plant physiology, 161(3), 1217-1228.

Garg, B., Puranik, S., Misra, S., Tripathi, B. N., & Prasad, M. (2013). Transcript profiling identifies novel transcripts with unknown functions as primary response components to osmotic stress in wheat (*Triticum aestivum* L.). Plant Cell, Tissue and Organ Culture (PCTOC), 113, 91-101.

Huang, X., Wang, G., Shen, Y., & Huang, Z. (2012). The wheat gene *TaST* can increase the salt tolerance of transgenic *Arabidopsis*. Plant cell reports, 31, 339-347.

Jiang, L., Zhang, D., Shao, Y., Yang, S., Li, T., Zhang, Z., & Li, C. (2012). Real-time quantitative PCR monitoring of antioxidant enzyme gene expression in wheat radicles treated with Cu^2+^ and Cd^2+^. Life Sci J, 9, 1679-1685.

Li, M., Ding, B., Wang, J., Yang, W., Wang, R., Bao, S., ... & Xie, X. (2013). Wheat TaWRKY10-1 is involved in biological responses to the salinity and osmostresses in transgenic *Arabidopsis* plants. Australian Journal of Crop Science, 7(6), 723-729.

Qin, Y., Wang, M., Tian, Y., He, W., Han, L., & Xia, G. (2012). Over-expression of *TaMYB33* encoding a novel wheat MYB transcription factor increases salt and drought tolerance in *Arabidopsis*. Molecular Biology Reports, 39, 7183-7192.

Ramezani, A., Niazi, A., Abolimoghadam, A. A., Zamani Babgohari, M., Deihimi, T., Ebrahimi, M., & Ebrahimie, E. (2013). Quantitative expression analysis of *TaSOS1* and *TaSOS4* genes in cultivated and wild wheat plants under salt stress. Molecular biotechnology, 53, 189-197.

Saqib, M., Zörb, C., Rengel, Z., & Schubert, S. (2005). The expression of the endogenous vacuolar Na+/H+ antiporters in roots and shoots correlates positively with the salt resistance of wheat (*Triticum aestivum* L.). Plant Science, 169(5), 959-965.
